# Expression of c-Jun, p73, Casp9, and N-ras in thymic epithelial tumors: relationship with the current WHO classification systems

**DOI:** 10.1186/1746-1596-7-120

**Published:** 2012-09-14

**Authors:** Yuqing Ma, Qiaoxin Li, Wenli Cui, Na Miao, Xia Liu, Wei Zhang, Chen Zhang, Jian Wang

**Affiliations:** 1 Department of Pathology, First Affiliated Hospital, Xinjiang Medical University, Urumqi, 830054, China; 2 Cancer Research Laboratory, Fudan University Shanghai Cancer Center, Shanghai, China; 3 Fifth Affiliated Hospital, Xinjiang Medical University, Urumqi, China; 4 Department of Pathology, Fudan University Shanghai Cancer Center, Shanghai, China

**Keywords:** Thymic tumour, Histologic classification, World Health Organization (WHO), c-Jun, p73, Casp-9, N-ras

## Abstract

**Background:**

To evaluate the expression and differential significance of c-Jun, p73, Casp-9 and N-ras in thymic epithelial tumors (TETs) with the aim to provide useful information for tumor biology and prospective therapy.

**Methods:**

In this study, we analyzed the expression of four chromosome 1-related genes, namely c-Jun, p73, Casp-9 and N-ras, in 60 cases of thymic epithelial tumors. The tumors included 52 thymomas and 8 thymic carcinomas which were categorized according to the current WHO classification systems.

**Results:**

Compared with the normal thymus tissue, all thymic epithelial tumors demonstrated higher expression of c-Jun and p73. The expression of c-Jun and p73 in type B2, B3 thymoma and thymic carcinomas was similar, and significantly higher than that in all other subtypes of thymomas. Unlike type A thymoma, the expression of Casp-9 was relatively lower in type B thymoma and thymic carcinomas. With respect to the clinical staging systems, c-Jun was more expressed in progressive tumors harboring higher stages. In contrast to c-Jun, p73 and Casp-9, there was no significant aberration with N-ras expression irrespective of either tissue or tumor types.

**Conclusions:**

The overexpression of c-Jun, p73 and Casp-9 in thymic epithelial tumors is closely related with the pathogenesis and biological behavior of the neoplasms. These candidate biomarkers provided useful information for prospective personalized therapy in the clinical management.

**Additional non-English language abstract language: Chinese:**

背景:评估c-Jun, p73, Casp-9 和 N-ras在胸腺上皮性肿瘤诊断和鉴别诊断中的运用.

方法:根据世界卫生组织最新的诊断标准60例胸腺上皮性肿瘤分类,运用Envision法检测c-Jun,p73,Casp-9 和N-ras在不同亚型肿瘤中的表达情况,并结合临床病理学特征进行分析.

结果:c-Jun和p73在肿瘤中的表达明显高于正常胸腺组织;c-Jun和p73在B3,B2型胸腺瘤和胸腺癌的表达类似,且表达明显高于其他类型的胸腺肿瘤;Caspase-9在B型胸腺瘤和胸腺癌中的表达相对低于A型胸腺瘤;c-Jun的表达更常见于高级别的胸腺肿瘤.

结论:c-Jun,p73和Casp-9在胸腺肿瘤中的表达很好地反映了肿瘤的生物学特点,为胸腺肿瘤的诊断和鉴别诊断提供了较好的理论基础.

**Virtual Slides:**

http://www.diagnosticpathology.diagnomx.eu/vs/1521774814749726

## Background

Thymic epithelial tumors (TETs) are a panel of rare neoplasms, located in anterior mediastinum, accounting for approximately 0.2-1.5% of all human malignancies [1]. TETs present with apparently distinctive histologic characteristics from other malignancies, however, a big challenge for further subtype to many general pathologists in the routine diagnosis [2]. Thymoma is one of the most common subtypes of TETs and consists with a spectrum of heterogeneous tumors presenting with thymic differentiation but differ in morphology and clinical behavior [3-5]. Based on the morphology, function and genetic features, thymoma was re-categorized into type A, AB, B1, B2, B3, and some other rare subtypes by World Health Organization (WHO) in 2004 [6]. It has been reported that type A and type AB thymoma employed with benign biological behavior, whereas type B thymoma presented with pernicious characteristics to some extent. Specifically, type B3 thymoma has a distinctively poor prognosis compared with other subtypes [7-9]. TETs subtypes closely related to the therapeutic schedules and prognosis of these diseases, however, reliable and rational methods for recognizing these subtypes are insufficient so far, if any.

Zettl *et al.*[10] declared that different TETs subtypes shared various recurrent genetic aberrations, and gain of chromosome 1 was the most common recurrent aberration (69%) in type B3 thymoma, which might be an attractive landmark for the clinical diagnosis. And the same results have been validated by the followed studies [11,12]. Evidences indicated that genes located in chromosome 1 were closely related to the initiation and progression of several human malignancies, *c-Jun* (1p32-31), *p73* (1p36.3), *Casp-9* (1p36.21), and *N-ras* (1p13.2) were such kind of genes, which might involved in the process of origination, proliferation, differentiation, and apoptosis of the malignant cell [13-17]. However, few studies were reported in TETs. Based on a clinicopathologic analysis of 80 cases with immunohistochemical reaction, Moran *et al*. indicated that the behavior of primary thymic neuroendocrine carcinomas seems correlated with tumor differentiation [18]. Alexiev *et al*. declared that autoimmune related disorders of thymoma contained with a significant population of CD20+ intratumoral B lymphocytes, and strong CD57 expresssion in thymomas may indicated with a concomitant neuromuscular disorder [19]. Besides, It was reported that a combined therapy may be considered as an another promising option (e.g. COX-2 inhibitors plus anti-EGFR antibody), especially when established chemotherapeutic schemes did no work[20].

To our knowledge, the combination of expression of c-Jun, p73, Casp-9, and N-ras was firstly evaluated in different subtypes of thymic epithelium tumors and normal thymus tissue. By trying to investigate the expression characteristics of those antibodies, we aim to build an efficient panel of biomarkers for clinical differential diagnosing between subtypes of TETs.

## Materials and methods

TETs cases included were formaldehyde-fixed, paraffin-embedded (FFPE) archival samples from the Department of Pathology in First Affiliated Hospital of Xinjiang Medical University between January 2001 and February 2010. All of the archival slides were reviewed by two independent senior pathologists (YQM and QXL) according to the latest WHO criteria, and any discrepancy between these two investigators was resolved with a third reviewer (WZ) in order to reach an ultimate decision on all of the items. Finally, 60 cases were recruited based on the following criteria: 1) pathologically confirmed TETs (thymoma and thymic carcinoma), 2) integrated clinicopathological information, 3) without any chemotherapy/radiotherapy performed prior to recruitment. Among those, including 26 male and 34 female (1:1.3) with an average age of 48.5 years (range 25–73 years). In addition, 11 normal biopsy thymic tissues were used as normal control (provided by Teaching and Research Office of Pathology, Basic Medical Academy of Xinjiang Medical University). Informed consent was obtained from all of the case and control subjects. All specimens were handled and approved by the hospital’s ethics committee.

Information of the total TETs was extracted based on the criteria from the CAP website data (http://www.cap.org > cancer protocols > thorax > thymoma and thymic carcinoma). Briefly, information of specimen integrity, histologic subtypes, regional lymph nodes, tumor extension, and procedure treatment were obtained from the surgical document, if any. Pathologic staging for thymomas according to Modified Masaoka Stage system; and staging for thymic carcinomas according to pTNM system [6].

### Reagents and Immunohistochemistry

The antibodies included: c-Jun (mouse monoclonal anti-human antibody sc-1694; 1:60; nucleus; Jingqiao Zhong Shan Biotechnology, Beijing, China); p73 (mouse monoclonal anti-human antibody; 1:60; cytoplasm; Boshide Biotechnology Wuhan, China); Casp-9 (mouse monoclonal anti-human antibody MCH6; 1:60; cytoplasm; Boshide Biotechnology Wuhan, China) and N-ras (mouse monoclonal anti-human antibody; 1:100; cytoplasm; Boshide Biotechnology Wuhan, China).

Experimental procedures were performed as previously described [21]. Briefly, serial 3-μm sections from formalin-fixed, paraffin-embedded tissues were collected onto poly-L-lysine coated slides and processed with a standard manual streptavidin peroxidase technique using a biotin-free detection system (Dakao, Colorado, USA) after a heat-induced antigen retrieval procedure. Ready-to-use Kit (EnVisionTM, Dakao, Colorado, USA) was used to visualize tissue antigens according to manufacturer’s instructions. Positive, Negative, and blank control was routinely performed.

### Immunohistochemical evaluation

Immunoreactivity was assessed by two senior pathologist (YQM and XL) who were blinded to clinicopathologic data, and any disagreements were resolved with a third reviewer (WZ) using a multi-headed microscope. Scoring of immunohistochemistry was based on two parameters: intensity of immunoreactivity and the exact location of immunoreaction. The immunostaining intensity was scored using the following semi-quantitative scale: 1) -, no reactivity (no staining or weak staining less than 5% of the target cells), 2) +, cases presented specific staining of more than 5% of the target cells, regardless of staining intensity, were scored as positive for c-Jun, p73, Casp-9 or N-ras[22].

### Statistical Analysis

Statistical analysis was performed using SPSS version 13.0. Group comparisons of categorical variables were evaluated using the Fisher’s exact or Pearson’s chi-square test. All *P*-values were two-sided, *P*-values less than 0.05 were considered to be statistically significant, less than 0.01 meant highly significant.

## Results

### Clinicopathologic results

Among the total 60 TETs, most of which involved anterior mediastinum (58/60), only one of these TETs involved superior mediastinum and right mid-lower mediastinum, respectively. The maximum diameters of the samples in our study ranged from 1.8 to 14.0 cm (average, 6.14 cm), among those, two type A, 19 type AB, four B1, 14 B2, 11 B3, two metaplastic thymoma, and eight thymic carcinoma were observed(seven primary squamous cell carcinomas, and one primary well-differentiated neuroendocrine carcinoma). 21 TETs cases employed an uninvolved-margin, as well as 39 margins involved by tumor. Concerning to the staging information, among 52 thymomas, there were 21 stage I, 19 stage II, 11 stage III, and one stage IV based on the Modified Masaoka Stage system; among eight thymic carcinomas, there were one T_2_N_0_M_0_, four T_3_N_0_M_0_, two T_4_N_0_M_0_ and one T_4_N_1_M_0_ based on the pTNM system; however, in order to obtained a powerful statistic results, we transform pTNM of thymic carcinoma into Mosaoka tumor stages based on comparison of Masaoka tumour stages and corresponding TNM classification [6], and the final results were 21 stage I, 20 stage II, 15 stage III, four stage IV based on the Modified Masaoka Stage system. No regional lymph node metastasis was found excepted for only one thymic carcinoma. No lymph-vascular invasion was observed in the current study. Most of the patients (48/60) saw a doctor due to chest pain and cough, in which 17 of the patients (28.3%) had myasthenia gravis (one of type A, four of type AB, eight of type B2, and four of Type B3). However, the remainder cases were asymptomatic and found by routine physical examination. Follow up data were available for 14 patients only [14], among which five of type AB, two of type B1, three of B2, one of B3, one of metaplastic thymoma and two of thymic carcinoma. Post-operative follow-up range from two months to 84 months, during the follow-up period, all cases were still alive, three cases (two of B2, one of AB) recur ptosis after operation and radiotherapy.

### Immunohistochemistry results

**Figure 1:**
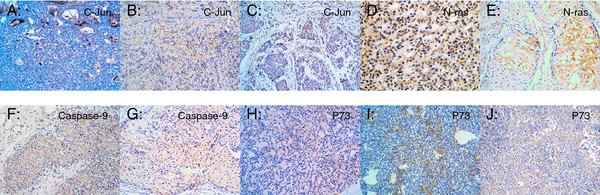
**c-Jun, N-ras, Caspase-9 and p73 immunoreactivity in TETs.A**-**C**: c-Jun staining in typeAB, B3 and thymic carcinoma; **D**,**E**: N-ras staining in typeA and B3; **F**,**G**: Caspase-9 staining in typeB2 and B3; **H**-**J**: p73 staining in typeA, AB and B2. EnVision, Scale bar: 50 μm Olympus CX51. photoshop software (PS).

In order to evaluate the diagnostic significance of c-Jun, p73, Casp-9, and N-ras expression in the distinction of TETs, we detected these markers with immunohistochemistry. The results of immunohistochemistry were seen in Figure 1.

c-Jun expression: The expression of c-Jun was mainly located in nucleus in the tumorous epithelium of thymoma. Statistically, it was found that expression of c-Jun in TETs was significantly higher than that in normal thymus tissue (*P <* 0.05, Table 1). Furthermore, statistical significant differences of c-Jun expression between subtypes were observed (*P <* 0.05), either. Thymic carcinoma , Type B3 and Type B2 thymoma ranked the first higher expression rate of c-Jun; they were 87.5% (7/8), 45.5% (5/11), and 42.9% (6/14), respectively. However, immunoreactions were not seen in Type A, B1, and metaplastic thymoma (Table 1). A statistical significant result between c-Jun expression and various clinical stages of TETs were found (*P <* 0.05), c-Jun expression were definitely higher in high stage (III + IV) when compared with the low stage (I + II) TETs (*P <* 0.01). However, no statistical discrepancy was observed in stage I *vs.* II, as well as stage III *vs.* IV, respectively (*P >* 0.05) (Table 2).

**Table 1:** Distribution of c-Jun, N-ras, Caspase9 and p73 expression in different subtypes of thymic epithelial tumors (TETs) n (%)

	**Type-A**	**Type-AB**	**Type-B1**	**Type-B2**	**Type-B3**	**Tca**^**a**^	**MT**^**b**^	**Total**	**NT**^**c**^	***P***^***d***^	***P***^**e**^	***P***^**f**^
	**N = 2**	**N = 19**	**N = 4**	**N = 14**	**N = 11**	**N = 8**	**N = 2**	**N = 60**	**N = 11**			
**C-Jun**	0 (0.0)	4 (21.0)	0 (0.0)	6 (42.9)	5 (45.5)	7 (87.5)	0 (0.0)	22 (36.7)	0 (0)	**0.011**	**0.014**	**0.002**
**N-ras**	1 (50.0)	3 (15.8)	0 (0.0)	1 (7.1)	5 (45.4)	2 (25.0)	0 (0.0)	12 (20.0)	3 (27.3)	0.187	0.888	0.364
**Caspase9**	2 (100.0)	5 (26.3)	0 (0.0)	10 (71.4)	7 (63.6)	4 (50.0)	2(100.0)	30 (50.0)	7 (63.6)	**0.016**	0.780	**0.019**
**p73**	1 (50.0)	5 (35.7)	0 (0.0)	9 (64.3)	8 (81.8)	4 (50.0)	1 (50.0)	28 (46.6)	1 (9.1)	**0.044**	**0.046**	**0.004**

Tca^a^: thymic carcinoma.

MT^b^: metaplastic thymoma.

NT^c^: normal thymic tissue.

*P*^d^: Two-sided Fisher’s exact test for distributions between different histologic subtypes of thymoma.

*P*^e^: Two-sided Fisher’s exact test for distributions between cases and controls.

*P*^f^: Two-sided Pearson’s chi square test for distributions between lower-grade TETs (type-A, AB, B1,MT) and higher-grade TETs (type-B2, B3, Tca).

**Table 2:** Different expression of c-Jun, N-ras, Caspase9 and p73 in different clinical stages of thymoma n (%)

		**Stage I N = 21**	**Stage II N = 20**	**Stage III N = 15**	**Stage IV N = 4**	**Total N = 60**	***P***^***a***^	***P***^***b***^	***P***^***c***^	***P***^***d***^
**C-Jun**	**+**	6 (28.6)	4 (20.0)	8 (53.3)	4 (100.0)	22 (36.7)	**0.009**	**0.004**	0.523	0.245
**N-ras**	**+**	3 (14.3)	3 (15.0)	6 (40.0)	0 (0)	12 (20.0)	0.184			
**Caspase9**	**+**	8 (38.1)	11 (55.0)	8 (53.3)	3 (75.0)	30 (50.0)	0.482			
**p73**	**+**	6 (28.6)	10 (50.0)	9 (60.0)	3 (75.0)	28(46.6)	0.089			

*P*^*a*^: Two-sided Fisher’s exact test for distributions between different stages of TETs.

*P*^*b*^: Two-sided Fisher’s exact test for distributions between stage III + IV and stage I + II of TETs.

*P*^*c*^: Two-sided Fisher’s exact test for distributions between stage I and stage II of TETs.

*P*^*d*^: Two-sided Fisher’s exact test for distributions between stage III and stage IV of TETs.

Caspase-9 expression: Similar Caspase-9 expression was observed both in thymic epithelium tumors and normal tissue, no statistics difference between them was observed (*P >* 0.05, Table 1). And significant difference of Caspase-9 expression among different subtypes of thymic epithelium tumors (*P <* 0.05) was observed, as showed in Table 1, almost all of the type A and metaplastic thymoma expressed Caspase-9 antibody, whereas none of the type B1 thymoma positive expression was observed. What’s more, an increasing immunoreactivity along with the higher clinical stages was observed, they were 38.1% (Stage I), 55.0% (Stage II), 53.3% (Stage III), and 75.0% (Stage IV), respectively, however, no statistically discrepancy was observed between stages (*P >* 0.05, Table 2).

P73 expression: Over-expression of p73 in thymic epithelium tumors was observed, and presented significantly discrepancy when compared with normal tissue (*P <* 0.05), as well as compared among subtypes (*P <* 0.05). The respective expression of p73 in type B3, type B2 and type B1 were 72.2% (8/11), 64.3% (9/14), and 0% (0/4), as showed in Table 1. It was indicated that type B3 has a significantly high expression of p73 than non-type B3 thymomas (*P <* 0.05). Meanwhile, there was no significant difference of p73 expression between type B3, B2 thymoma and thymic carcinoma (*P*>0.05, data not shown). p73 positive expression in different clinical stages of thymoma was 38.1% (Stage I), 55.0% (Stage II), 53.3% (Stage III), and 75.0% (Stage IV), respectively. No statically significant among different clinical stages of p73 expression was observed (*P >* 0.05, Table 2).

N-ras expression: There was no significant difference of N-ras expression between thymic epithelium tumors and normal tissue controls (*P >* 0.05) (Table 1), similar negative results were observed among different subtypes of TETs (*P >* 0.05, Table 1). N-ras positive rates of different clinical stages of thymic epithelium tumors were 14.3% (Stage I), 15.0% (Stage II), 40.0% (Stage III), and 0% (Stage IV), respectively, among which no significant difference of N-ras expression was found (*P >* 0.05, Table 2).

We also evaluated the expression distribution of those four antibodies between low-grade TETs (including type-A, type-AB, typeB1, and metaplastic thymoma) and high-grade TETs (including type-B2, type-B3, and thymic carcinoma), and found that c-Jun, Caspase-9, and p73 expression was statistically significant with high-grade TETs, the *P*-values were 0.002, 0.019, and 0.004, respectively. No statistically significant discrepancy was found between the expression of those antibodies and some other clinicopathological characteristics.

## Discussion

Thymomas were neoplasms arising from or exhibiting differentiation toward thymic epithelial cells. It has been reported that different subtypes of thymoma have different genetic characteristics, recent studies indicated that chromosomal 1 gain plays an important role in molecular genetic mechanism of thymic epithelium tumors [10,23-25].

C-Jun (cellular Jun), a member of nucleus transcription factor, is an oncogene locating on chromosome 1. It was indicated that the expression of c-Jun immunohistochemistry can reveal the mRNA level of c-Jun [23]. In this project, expression of c-Jun in 22 from 60 (36.7%) TETs were observed, which specifically located on cell nucleus. Statistical test showed that the abnormal expression of c-Jun was significantly higher in thymic epithelium tumors than that in normal tissue controls. There were also statistical differences of c-Jun positive expression in different subtypes of thymoma. Among those TETs, including thymic carcinomas, type B3, and type B2 thymomas took the higher percentage immune reaction (more than 40%) of c-Jun. Our results were consistent with Sasaki’s research [23], indicating a strong positive expression of c-Jun might correlate with high grade TETs. Therefore, we speculated that c-Jun might be regard as a potential positive regulator of cell reproduction [26,27], or might play an important role in the process of tumor differentiation. Besides, proliferation index Ki-67 was increase in type B3 thymoma cells[28]. In our project, there were statistical differences of c-Jun expression in different clinical stages of thymic epithelium tumors: advanced thymomas (III + IV) were significantly higher than those of the early thymomas (I + II); and there was no statistical difference between stage I and II; neither between stage III and stage IV. This research showed that the expression of c-Jun increased in invasive thymic tumors, which also suggested that c-Jun might be used to help judging the biological behaviors, clinical stage, and prognosis of tumors.

N-ras is one of the ras gene family members locating on chromosome 1. N-ras function as an important factor in the process of cell proliferation, senescence, immortalization and carcinogenesis. N-ras can also inhibit the cancer cells proliferation by Suv39h1 and H3K9 methylation. Mutational ras protein can affect cell proliferation, cell cycle regulation and anti-apoptotic signal by decreasing the activity of endogenous GTPase, or transcriptional decreasing the expression of Fas receptor and regulating the last time of the p38 activity of Jun N-terminal protein kinase (JNK) by Ral-GEF (Ras related GTPase-guanine exchange factor) pathway [29] Meanwhile, N-ras has different function to the generation of cancer in different individuals[30-32]. In our study, N-ras expression was found in 12 cases of TETs, however, no significant difference was observed between thymic epithelium and normal tissue controls, similar results existed between the subtypes of thymic epithelium tumors. The results suggested that N-ras might play a role in the generation of thymic epithelium tumors. However, it will be hard to indicate the relationship of N-ras expression in different subtypes of thymic epithelium tumors due to the small sample size of the current study. Further studies are needed to confirm these observations and to determine the mechanism of N-ras in the origin and development of TETs. No statistical differences were detected in N-ras expression of different clinical stages of thymic epithelium tumors.

Caspase-9 gene locates on 1p36.3-p36.1. It precipitates in mitochondria induced cell senescence pathway [32]. Several studies indicated that decreasing Caspase-9 transcription and translation are detected in head and neck squamous cell carcinoma [33], and leukemia [34]. In our project, 30 out of 60 (50%) thymic epithelial neoplasms have positive Caspase-9 expression, which was slightly lower than the Caspase-9 expression in normal control tissues (7/11, 63.6%). But the difference was not statistically significant. The result indicated that there were significant differences among Caspase-9 over expressions in different subtypes of thymic epithelium tumors. In different subtype, the expression of Caspase-9 in thymic epithelium tumors mainly existed in thymomas constructed by bland epithelial cells, including type A and metaplastic thymoma. Caspase-9 presented a lower expression in type B thymoma and thymic carcinoma than in type A and metaplastic thymoma, which was consistent with previous research [33,34]. The decreasing tendency of Caspase-9 transcription and translation indicated that the interruption of Caspase-9 related apoptosis signaling pathways might promote the generation of type B thymoma and thymic carcinoma. However, in the current study, we first divided TETs into two groups as described above (low-grade TETs and high-grade TETs), and found that Caspase-9 presented relatively lower expression in low-grade TETs when compared with that in high-grade TETs. The mechanism was not clear, and need more future studies to validate our results. No statistical differences are detected in Caspase-9 expression of different clinical stages of thymic epithelium tumors.

p73 gene locates on human chromosome 1p36.33. Many isomers of p73 were identified, and the expression and interaction of those different isomers involved the process of regulate transcription and growth inhibition [35,36]. The fact that p73 abnormal expression was observed more common in cancer tissues than in normal tissue indicated that p73 might be an oncogene [37]. In this project, over-expression of p73 in thymic epithelium tumors was significantly higher than that in normal control tissue. This result suggested that the expression of p73 increased in thymic epithelium neoplasm, which was similar with the previous research on digestive system tumor [35]. The p73 protein detected by immunohistochemical methods were probably wild type. The positive expression of p73 in different subtypes of thymic epithelium tumors existed statistical differences. In addition, our results indicated that p73 presented with similar positive expression levels in type B2, B3 thymoma and thymic carcinoma, which were significantly higher than other subtypes of thymoma. This conclusion suggested that p73 might play an important role in type B2, B3 thymoma and thymic carcinoma. It was also revealed that the molecular change of type B2, B3 thymoma might be similar with thymic carcinoma, and differ from other types of thymoma. Considering previous research results of our group that p53 protein positive expression increased in type B3 thymoma [28], it can be inferred that p73 and p53 protein mutants might embrace a synergistic effect in thymoma. However, the limitation of the follow-up data was too small to make such conclusion, the further analysis between p73 expression and prognosis need more data from the future follow-up data.

In summary, the results indicated that c-Jun and p73 expressed significantly higher in thymic epithelium tumors than in normal control tissues. c-Jun and p73 also had similar positive expression level in high-grade TETs, which is significantly higher than low-grade TETs. In addition, Caspase-9 expression was relative lower in type B thymoma and thymic carcinoma. However, no significant difference of N-ras expression among different tissues of the thymus and different thymic epithelium tumors was observed. What we observed suggested that different genes on chromosome 1 might employ different functions in the generation and development of thymic epithelium tumors. c-Jun and p73 may promote the tumor formation. Previous studies of our group also suggested that chromosome 1 gain was significantly higher in thymic epithelium tumors than normal thymus tissue, and it was also higher in type B3 thymic epithelium tumors than other subtypes of thymoma [11]. It is highly possible that type B3 thymoma has a different molecular change with other types of thymoma, and similar with thymic carcinoma. Those evidences suggested that type B3 thymoma should be distinguished from other subtypes of thymoma and might be classified as a intermediate malignant tumor, which needs more future studies to validate our results before they can have widespread application. Meanwhile, The use of a combination of c-Jun,p73 and Caspase-9 could help differential diagnosing.

## Abbreviations

TETs: Thymic epithelial tumors; WHO: World health organization; FFPE: Formaldehyde-fixed, paraffin-embedded.

## Competing interests

The authors declare that they have no competing interests.

## Authors’ contributions

Ma Y. participated in the design of the study and given final approval of the version to be published. Li Q. participated in its design and coordination and helped to draft the manuscript. Cui W. performed the statistical analysis. Miao N. carried out the immunohistochemistry method. Liu X. was response for the quality of the immunohistochemistry result. Zhang W. was response for the quality of the study. Zhang C. helped to draft the manuscript. Wang J. participated in the design of the study and performed the statistical analysis. All authors read and approved the final manuscript.

## Funding

This study was supported by the grant from “Project of the Foundation and Application of the Comprehensive Pathologic Diagnostic Platform” Recruitment at technology program of Xinjiang Uygur Autonomous Region (201233142).
